# Observations on Digitalis, with Cases of Consumption and Hydrothorax

**Published:** 1806-09

**Authors:** 


### Observations on Digitalis, with Cases of Consumption and Hydrothorax. By Dr. Hamilton.

*To the Editors of the Medical and Physical Journal.*

Gentlemen,

THE pages of your Journal have ceased to exhibit the high wrought encomiums on digitalis, which a few years ago so amply contributed to swell the hopes of your readers, and of their consumptive patients; and I fear the efficacious use of this plant has nearly, in an equal degree, been banished from practice. Were such the fate of medical theories alone, there would perhaps be little room for regret. But it is much to be lamented, that many *medi-*

 *cal*

*cal facts* stand in the like predicament, and that remedies recommended as infallible, in relieving and curing the most obstinate and dangerous disorders, even by the first authorities, are frequently, when experience has decided upon their merits, consigned to oblivion. Such has, in a great degree, been the fate of the digitalis; nor is there any medicine concerning which, the opinion of physicians has more fluctuated. To the high expectations raised of it, by the respectability, and confidence in its powers, of the Gentlemen who brought it into notice, as a remedy for consumption, no doubt, much of its present neglect is owing; for in many such cases, it was found entirely to fail, even when exhibited in the most skilful and persevering manner; and it was consequently condemned, as inadequate to the cure of any, because it had been asserted of it, that it was able to effect the cure of all. But this remedy has not been unmeritedly neglected on this account alone; the manner in which it is usually collected, prepared, and exhibited, having, in at least an equal degree, contributed to its disuse. Had Dr. Drake's excellent observations on these particulars been duly regarded, it would, long ere this, have been found capable in many cases of producing the most salutary effects, and its efficacious but guarded exhibition would have become general. In many of the cases in which I found the best effects from this medicine, I have been told, it had been fully tried. These trials have usually been confined to the exhibition of two spoonsful, of a half pint mixture, containing 20 or at most 30 drops of the tincture, or a grain of the powder, night and morning; both preparations commonly obtained from a London druggist. In Mr. Tilney's case of Hydrothorax, related below, half a grain of the powder was ordered by a very eminent physician, night and morning; and when I proposed it, in another case, to which I thought its use eminently adapted, to a physician, high in the medical department of the army, he informed me, he had twice been prevailed upon to sanction the exhibition of digitalis, but that nothing should ever induce him to prescribe it again. Thus has this powerful and excellent remedy been trifled with, and neglected.

Before proceeding to relate the experience I have had, of the use of foxglove in consumption, and hydrothorax, it may be proper to state in a few words the manner of preparing it, as this will shew that it may be procured with very little trouble, and in any situation.

I cultivate the plant in my garden, where by common attention, attention, it flourishes exceedingly, and I find that it grows with equal luxuriance in the grounds of several of my friends, although the situation and soil may considerably differ; nor have I perceived any deficiency, in the quality of the leaves procured from these different situations. My largest collection, however, has for some years been made, in the shrubberies of Lord Rous, at Henham, which enjoy an elevated situation, a few miles removed from the sea, and a gravelly soil. I collect the largest leaves from the most vigorous and healthy plants, when in full flower, and dry them in the shade. When this is effected, I rub them into coarse powder, taking out the large stems, and preserve them in well stopped bottles. The powder thus prepared is of a fine green colour, and has a fragrant smell, not unlike new made hay, but stronger.

Since Dr. Drake directed the attention of the Faculty to the employment of this remedy in consumption, by which he has conferred upon the healing art a great benefit, I have used it in very many of the cases that have fallen under my care. In the early stages of that disorder, I think I cannot be mistaken in supposing, that I have often seen it suspend, and sometimes avert, its commonly fatal termination. And in more advanced cases, where such advantages could not be hoped for, it has almost invariably succeeded in alleviating the most distressing symptoms, when by its gradual increase, a *reduction of the velocity of the pulse could be effected.* Speaking of this effect of digitalis, Dr. Ferriar says, "If any man had expressed an opinion a few years ago, that we should discover a medicine capable of reducing the pulse, without danger, from 120 in a minute to 75 or 80, at the will of the practitioner, he would have been ridiculed as a visionary. Such, however, under proper management, is the power of digitalis." Dr. Currie also observes, "this medicine may almost be said to be possessed of a charm for allaying inordinate action of the heart and arteries." Were this effect of the foxglove as constantly to be obtained from its exhibition, as these eminent physicians, and others, have supposed, I can have no doubt that it would prove, in the early stages of a disease, the danger of which may for the most part be measured by the rapidity of the pulse, as efficacious as its most sanguine admirers have hoped. But I apprehend the reduction of the circulation is very far from a constant effect of the most guarded use of this remedy. In much the larger number of the patients, to whom I have given it, in consumption, in saturated tincture, ture, and in the most gradually increased doses, I have been compelled to abandon its use, before the circulation was reduced, from its generally debilitating effects, from nausea and sickness, or affections of the head, by which its farther use was prohibited. This disappointment I think I have observed to hold a pretty equal pace with the tendency to inflammatory action that was present. When there has been great heat, flushing of the cheeks, thirst, acute pain in the side, and the symptoms from which active inflammation is concluded to be present,—till these symptoms were removed, I have generally failed in reducing the pulse. In pneumonia also, and in other diseases of active inflammation, I have never seen good effects from digitalis, till this disposition was lessened by the use of the lancet, &c.; and until the expectoration had become copious, then, as well as in vomica, it has appeared very beneficial. I regret the necessity these observations lay me under, of differing in opinion from such a man as Dr. Currie, who says, "under the precautions pointed out by Dr. Withering, without the strictest attention to which no practitioner should prescribe this singular and powerful medicine, I have employed the digitalis to a very considerable extent, in inflammations of the brain, of the heart, and of the lungs; and have succeeded with it in situations where I should otherwise have despaired. I have also found it an excellent remedy in inflammatory rheumatism."

The Doctor however observes, "Digitalis does not, indeed, supersede the use of the lancet in these diseases, but it diminishes the extent to which it is required; and it may be used with safety and success, in cases where the lancet can no longer be employed." I have, however, stated only what I have repeatedly seen, and what many of my medical friends have witnessed.

When it has been apprehended from the presence of the symptoms above mentioned, that the digitalis would not produce the desired effect of lessening the frequency of the pulse, I have put the patient on a diet of milk and vegetables, prescribed saline and aperient medicines, and even have had recourse to moderate bleeding, before the tincture was given; and it has, after these precautions, produced every effect I expected from it. The following case will illustrate some of the preceding remarks, and I think fully establish the power of the foxglove over a strong disposition, at least, to phthisis; many others, shewing a greater or less degree of the same power, might be given, but as I chiefly wish to solicit the attention of your readers to to the efficacy of digitalis over hydrothorax, I shall content myself with this.

A. B. a female of delicate habit of the consumptive age, was in the beginning of August, 1805, affected with pain in the region of the heart; rigors, alternating with heat, slight cough, with difficulty and oppression of breathing, and quickened circulation. She continued under these symptoms some days, and when I saw her, the pulse was increased to 120. I directed her to lose a small quantity of blood, and to apply a blister to the side; she omitted the use of wine and meat, and lived chiefly on vegetables and fruit. By this plan, in a few days, she was in some measure relieved, but the pulse continuing as rapid as ever, she began the use of the tincture of digitalis, in a saline draught, in doses of ten drops every eighth hour, and it was increased one drop in each dose, daily, till she had taken seventeen drops at a time. The pulse was now 85, and without any farther increase of the dose, it was in a fortnight, from its first exhibition, reduced to 65. At this rate it was kept for the space of a month longer, by the occasional use of the medicine, with the decided amendment of all the symptoms; and it was hoped that no further anxiety need be entertained respecting the event of the case. About the middle of September, however, I was desired to visit this patient again, and I found her labouring under very nearly the same symptoms as at first, but the pain in the chest and difficulty of breathing were less urgent, and no evacuation was now judged necessary; the digitalis was prescribed as before, and acted in the same *kindly* manner; the pulse was reduced, and every symptom was again alleviated. In October, the complaint appeared again to make fresh progress, and it was deemed necessary that the patient should go to London for the benefit of additional advice, and with the intention of proceeding to a warmer situation, should that be recommended. A few days after her arrival there, I met Dr. Baillie in consultation. This gentleman judiciously observed, that the medicine to which she owed so much, possessed not, nevertheless, the power of preventing the return of her disorder, as had been already proved. And he advised the uva ursi, as recommended by Dr. Bourne, a remedy from which, in much larger doses than given by him, I think I have in hectic cases, seen considerable advantage. Here, however, it was not productive of benefit, and in a few days Dr. Baillie advised her to recur to the digitalis again; and its use was a third time attended by the the same happy effects as before. Since that period, she has been obliged to have recourse to the tincture every eight or ten weeks, and has now been placed seven times completely under its influence; and it has never failed to relieve her from the urgency of the symptoms that first required its use. For many weeks past, it has not been taken, as the pulse has never amounted to more than 80, and no symptom of the former complaint has appeared; flesh and strength also are acquired, and there seems no well grounded fear that the former appearances of disease will return. The progress of the case, however, will be carefully watched, and the digitalis, should it be necessary, again resorted to.

Of the nature of this case, and its tendency to consumption, I presume no doubt will be entertained. Its apparent danger is proved by the following extract of a letter from Dr. Baillie, (whose accurate judgment is doubted by none) dated January, 1806.

"I have seen our patient this day, after an interval of six weeks; she does not seem to have lost flesh or strength, yet the disease has made evident progress, as some expectoration has been observed, and I can now have no doubt of its fatal termination."

Nor can there, I think, be any hesitation in ascribing the recovery to the Fox-glove. Of any person inclined to deny the beneficial influence of this remedy, in this instance, I would beg to ask, what would have been the effect of allowing the pulse to continue at 120 for even one month? In the foregoing remarks upon the influence of the digitalis upon consumption, I have stated what I have seen, and what I think will be found by others, who exhibit it with attention to Dr. Drake's directions, and without prejudice. In a great majority of instances, I fear it will be found to fail entirely, or to produce only temporary benefit; in some it will, undoubtedly, in the early stages, suspend the progress of the disease, and avert its fatal termination, and in several it will afford relief, to be obtained from no other source. In hydrothorax, a disorder generally supposed equally fatal, its curative powers are much more certain; and where organic affections are not present, or the frame is not entirely worn out by age and long protraction of disease, it seems to me but a short way removed from the nature of a specific, intending to express by that term, a remedy that is generally successful.

(To be concluded in our next.)

*Halesworth, July* 14, 1806.

